# Successful long-term *in vitro* cultivation of *Treponema pallidum* subsp. *pertenue* strains of human and non-human primate origin

**DOI:** 10.1080/22221751.2026.2751730

**Published:** 2026-10-17

**Authors:** Juraj Bosák, Matěj Hrala, Lenka Mikalová, Petr Andrla, Petra Pospíšilová, Klára Janečková, Kateřina Hanáková, David Potěšil, Zbyněk Zdráhal, David Šmajs

**Affiliations:** a Department of Biology, Faculty of Medicine, Masaryk University, Brno, Czech Republic; b Central European Institute of Technology, Masaryk University, Brno, Czech Republic; c National Centre for Biomolecular Research, Faculty of Science, Masaryk University, Brno, Czech Republic

**Keywords:** *Treponema pallidum*, TPE, yaws, *in vitro* cultivation, proteome

## Abstract

The long-term *in vitro* cultivation system for *Treponema pallidum* enabled *in vitro* propagation of several historical and contemporary *T. pallidum* subsp. *pallidum* (TPA) strains as well as *T. pallidum* subsp. *endemicum* (TEN) strain Bosnia A. In contrast, the long-term *in vitro* cultivation of *T. pallidum* subsp. *pertenue* (TPE) remained challenging. In this study, we demonstrate the first successful long-term *in vitro* cultivation of TPE strains including CDC-2, a human yaws isolate, and strain Fribourg-Blanc, a simian isolate. The *in vitro* cultivation of TPE Gauthier (a human yaws isolate) was positive for presence of treponemes/treponemal DNA for 24 weeks; however, the Gauthier growth rate was insufficient for long-term stable cultivation. In accordance with findings from long-term cultivation, binary co-cultivation with the TPA strain DAL-1 (generation time: 33 hours) revealed generation times of 41.7 hours for CDC-2 and 49.0 hours for Fribourg-Blanc. Furthermore, the first descriptive proteomic characterization of TPE strains CDC-2 (776 detected proteins; 72.7% of annotated proteins) and Fribourg-Blanc (694 detected proteins; 65.2% of annotated proteins), as well as TEN strain Bosnia A (889 proteins; 83.6% of annotated proteins), has been achieved from *in vitro* cultures. The established *in vitro* cultivation of TPE provides an important tool to advance studies focused on yaws pathogenesis and non-human primate infections.

## Introduction

Human pathogenic treponemes related to *Treponema pallidum* belong to three subspecies responsible for distinct diseases: syphilis (subsp. *pallidum*, TPA), yaws (subsp. *pertenue*, TPE), and bejel (subsp. *endemicum*, TEN). These subspecies share a high genomic similarity (>99.7%; [1,2]); however, infections caused by them differ significantly in clinical manifestations. While syphilis is a venereal disease with a global impact, yaws and bejel are endemic treponematoses, which are characterized as non-venereal, skin/mucosa-limited, or bone-affecting diseases typically found in tropical and arid regions, respectively [3].

For decades, these pathogenic treponemes were considered uncultivable and were propagated via experimental animal infection. While the rabbit serves as a universal host for all *T. pallidum* subspecies, hamsters efficiently support the propagation of TPE and TEN, whereas guinea pigs provide an additional model for TPA and TPE [4–6]. Due to the inherent difficulties of these animal models, only a limited number of pathogenic strains have been successfully isolated and maintained worldwide over the past several decades [7].

In 2018, a long-term *in vitro* cultivation system for *T. pallidum* was established [8], utilizing rabbit Sf1Ep cells and TpCM-2 medium in a low-oxygen atmosphere, along with weekly subcultures. The use of this technique enabled *in vitro* propagation of several historical TPA strains (Nichols, DAL-1, Madras, Haiti B, SS14, Mexico A, Philadelphia 1, Grady, UW231B, UW249B) [8–11], as well as contemporary TPA strains (MU1–MU6) [12]. While this *in vitro* system has also allowed cultivation of the TEN strain Bosnia A [9], the propagation of TPE strains has remained unsuccessful until now.

In this study, we report the first successful long-term *in vitro* cultivation of TPE strains, including the human yaws isolate CDC-2 and the simian isolate Fribourg-Blanc. In addition, we evaluate the human yaws isolate Gauthier, which showed treponemal survival for several weeks although its growth rate was insufficient for long-term stable propagation. Furthermore, we quantified their growth parameters in comparison with the TEN strain Bosnia A and the TPA strain DAL-1, and describe the *in vitro* proteomes of TPE strains CDC-2 and Fribourg-Blanc, as well as TEN strain Bosnia A.

## Methods

### Source of *T. pallidum* strains

The *T. pallidum* subsp. *pertenue* (TPE) strains Gauthier and CDC-2 were kindly provided by Dr. S. J. Norris (University of Texas Health Science Center, Houston), and the simian isolate Fribourg-Blanc was provided by Dr. D. Cox (Centers for Disease Control and Prevention, Atlanta). The *T. pallidum* subsp. *endemicum* (TEN) strain Bosnia A was also obtained from Dr. D. Cox (Centers for Disease Control and Prevention, Atlanta). The *T. pallidum* subsp. *pallidum* (TPA) strain DAL-1 was provided by Dr. D. Edmondson (University of Texas Health Science Center, Houston); following rabbit passage and subsequent *in vitro* cultivation, the resulting culture was designated as DAL-1, clone PV171 (Supplementary Table S1). The TPA strain SS14 was obtained from Dr. K. Hawley (University of Connecticut School of Medicine, Farmington). All strains were delivered as frozen suspensions derived from rabbit testes. For detailed characteristics of the *T. pallidum* strains, see Supplementary Table S1.

### *In vitro* culture of supporting cells

Sf1Ep cells (NBL-11) were kindly provided by Dr. D. Edmondson (University of Texas Health Science Center, Houston) and were originally purchased from the American Type Culture Collection (Rockville, MD; ATCC line CCL-68). Cultivation of rabbit Sf1Ep cells was performed as described previously [8]. Briefly, Sf1Ep cells were maintained in Eagle’s MEM (M4655, Sigma-Aldrich) supplemented with non-essential amino acids (1%; M7145, Sigma-Aldrich), L-glutamine (1%; G7513, Sigma-Aldrich), sodium pyruvate (1%; S8636, Sigma-Aldrich), and fetal bovine serum (10%; F4135, Sigma-Aldrich). Cultures were incubated at 37 °C in a humidified atmosphere containing 5% CO_2_. Sf1Ep cells were subcultured weekly (750,000 cells, T75 culture flask). Sf1Ep cells from passages 40 to 60 were used in this study.

### Long-term *in vitro* cultivation of *T. pallidum* strains

All *T. pallidum* strains were continuously cultivated *in vitro* using an original cultivation system [8] with a few modifications introduced in our laboratory to improve handling and stability of *in vitro* cultures. These modifications included a lower number of Sf1Ep cells and an increased inoculation dose of treponemes during subcultures [11]. Briefly, treponemes were cultivated in a well (6-well plate, 92406, Techno Plastic Products, Switzerland) containing TpCM-2 medium (4 mL) and a monolayer of rabbit cells Sf1Ep (50,000 cells). At seven-day intervals, the treponemal cultures were harvested using Trypsin-EDTA, (2 × 500 µL, 37°C, 5 min) and centrifuged for depletion of Sf1Ep cells (250 × g, 5 min). The supernatant containing treponemes (5 µL aliquot) was subjected immediately to dark-field microscopy (see below) and then inoculated into a freshly prepared well and cultivated at 34 ⁰C in a low oxygen atmosphere (2.5%) for 7 days. Additionally, an aliquot (200 μL) was frozen at −20 °C for subsequent PCR analysis (see below).

During the stable phase of cultivation, treponemes were typically passaged using 1 mL of the treponemal culture. When necessary, an enrichment protocol was employed [12]. This protocol involved the concentration of 4 mL of the treponemal suspension (10,000 × g, 5 min) to pellet the treponemes. The treponemal pellet was then resuspended in TpCM-2 medium (1 mL) and inoculated into a culture well.

Throughout the long-term cultivation, each *T. pallidum* strain was maintained in two independent wells as separate parallel cultures.

### Dark-field microscopy for the detection of treponemes

During subculturing, treponemal numbers and viability (i.e. motility) were investigated using dark-field microscopy (DFM). Briefly, each treponemal culture (5 μL) was examined using a BX53 microscope (Olympus Corporation) at 400× magnification, with five individual fields screened per sample. The number of treponemes was estimated based on the formula: one treponeme per field equals 340,000 treponemes per mL of culture [10]. During the enrichment phase of cultivation, the concentrated fraction of treponemes was analyzed by screening 50 individual fields, with a detection limit of 6,800 treponemes per millilitre.

### Nested PCR for the detection of treponemes

Total DNA was isolated from harvested treponemal cultures (200 μL) using QIAamp DNA Blood Mini kit (QIAGEN) following the Blood and Body Fluids protocol. DNA was eluted into AE buffer (100 μL). While the identity of TPE/TEN strains was determined by nested PCR targeting the TP0136 and TP0548 loci [13], nested PCR amplification of the TP0319 locus (with a detection limit of 390 treponemes/well, [12]) was used for the detection of treponemal DNA in DFM-negative samples. For detailed information regarding the primers and PCR conditions, see Supplementary Table S2.

### Quantification of *T. pallidum* growth differences using binary *in vitro* co-cultivation

To compare and quantify the *in vitro* growth rates of various treponemal strains, binary co-cultivation experiments were performed as described previously [11]. TPE strains (CDC-2 and Fribourg-Blanc) and the TEN strain Bosnia A were co-cultivated in three pairwise combinations among themselves and additionally with the TPA strain DAL-1 (another three combinations), which served as a reference for determining their generation times. For each pair, the low-dose inoculum consisted of a mixture containing 250,000 cells per strain, determined by dark-field microscopy and obtained from stable long-term cultures. These co-cultures were then maintained under standard conditions (34 ⁰C, 2.5% O_2_, 5.0% CO_2_), with subcultivation performed every 7 days using 500 µL of inoculum. The low inoculation dose and weekly subculturing ensured that the treponemes remained in the exponential growth phase throughout the experiment [8,11], which minimized alteration in growth rate that could affect the calculations. Moreover, TPE/TEN combinations were analyzed after 28 days (i.e. 4 subcultures) to ensure the precise determination of subtle growth differences, while binary co-cultivations involving DAL-1 were analyzed after 14 days (i.e. 2 subcultures), as DAL-1 completely outgrew the other strain at later time points, which precludes reliable quantification of generation times. The experiment was performed as a single biological replicate for each co-culture.

DNA was isolated from each co-cultivation well at the start and end timepoints (200 μL; QIAamp DNA Blood Mini Kit, QIAGEN). Ten genomic regions were selected to distinguish between the strains: strain-specific loci (TP0303, TP0346, TP0733, and TP0986), TPE/TEN-differentiating loci (TP0092, TP0457, and TP0864), and TPA-distinguishing loci (TP0164, TP0344, and TP0691). Consistent amplification was ensured by using identical primer binding sites across all strains, and results from seven different loci per co-culture were averaged to minimize potential locus-specific PCR variation (see Supplementary Table S3). Furthermore, amplification was performed using a high-fidelity polymerase (TaKaRa PrimeSTAR®) and a touchdown PCR protocol: 94 °C for 1 min; 8 cycles of 98 °C (10 s), 68–61 °C (15 s, decreasing 1 °C per cycle), and 68 °C (1 min 45 s); followed by 35 cycles of 98 °C (10 s), 61 °C (15 s), and 68 °C (1 min 45 s); with a final extension at 68 °C for 7 min. Each PCR reaction (25 µl) contained water (16.3 µL), 5x GLX buffer (5 µL), polymerase (0.5 µL), dNTPs (2 µL), primers (0.1 µL each, 100 pmol/µL), and isolated DNA (1 µL). Purified PCR products (QIAquick PCR Purification Kit, QIAGEN) were equimolarly pooled (∼1 × 10^10^ molecules per locus) to create 12 distinct mixtures representing two time points for each of the six binary combinations (see Supplementary Table S3). These pools were sequenced (150 bp paired-end reads, Illumina NovaSeq, Eurofins Genomics).

Bioinformatic analysis was performed as described previously [11,14]. Briefly, low-quality reads were filtered (FastQC v0.11.5 in conjunction with MultiQC v1.8; [15,16]) and adapters were trimmed (Fastp v0.20.1; [17]). The pre-processed reads were separately mapped against the reference sequences of both strains used in the co-cultivation (BWA MEM tool v0.7.17; [18]). The overall mapping quality was assessed (QualiMap v2.2.2; [19]) and variants specific for individual treponemal strains were identified (Freebayes v1.3.6; [20]).

The number of strain-specific reads at both time points was determined to calculate the relative growth ratio for each co-culture (i.e. the fold difference between strain ratios at the start and end points; Supplementary Table S4). Generation times of the TPE and TEN strains were estimated using the TPA strain DAL-1 as an internal growth standard (defined generation time of 33.0 h; [11]). During a 14-day period (corresponding to 2 subcultivation intervals of 7 days), the DAL-1 strain underwent 10.18 generations (n_DAL-1_ = 14 days × 24 h / 33 h). The difference in the number of generations (Δn) between the analyzed strain and DAL-1 was identified using the formula: Δn = log_2_(relative growth ratio). This value was then used to calculate the specific generation time for each TPE and TEN isolate by adjusting the reference number of generations (n_DAL1_ ±Δn) over the experimental period.

### Preparation of treponemal samples for proteomic analysis

Long-term *in vitro* cultures of TPE and TEN strains (i.e. CDC-2, Fribourg-Blanc, and Bosnia A) were used as the source of treponemes. Additionally, *in vitro* cultures of TPA strains DAL-1 and SS14 were included as representatives of the Nichols-like and SS14-like groups, respectively [21]. Treponemal samples were processed as previously described by Bosák et al. [22]. Briefly, a standardized inoculum for each strain was established in individual wells (4 × 10⁶ treponemes, 50,000 Sf1Ep cells, and 4 mL of TpCM-2 medium) and cultivated under standard conditions (34 °C, 2.5% O₂ atmosphere) for 7 days. The harvested treponemal culture (3 mL) was then inoculated into a T75 flask containing TpCM-2 medium (10 mL) and Sf1Ep cells (250,000). After another 7 days of cultivation, treponemes were harvested using a trypsin-free dissociation medium (4 mL, 34 °C, 2.5% O₂ atmosphere, 30 min [23]). Following Sf1Ep cell depletion (250 × g, 5 min), the treponemal suspension was collected (14 mL) and concentrated in PBS (10,000 × g for 5 min, repeated 5 times), resulting in a final volume of 200 μL. Treponemal counts were quantified by dark-field microscopy, and samples were immediately frozen (−20°C). The experiment was performed as a single biological replicate for each *T. pallidum* culture.

Based on dark-field microscopy, the treponemal numbers used for proteomic analysis were as follows: CDC-2 (0.4 × 10^6^), Fribourg-Blanc (0.13 × 10^6^), Bosnia A (1.7 × 10^6^), DAL-1 (17.0 × 10^6^), and SS14 (6.8 × 10^6^). Additionally, highly concentrated TPA strains were diluted 10- and 100-fold and used as controls to evaluate the reproducibility of proteomic analysis and the relevance of clustering in Principal Component Analysis (PCA, see below) with respect to the amount of treponemal input.

### Mass spectrometry (LC-MS/MS) analyses

For proteomic analysis, we employed our previously described protocol [22] with minor modifications. Briefly, *T. pallidum* samples (100 μL) were lysed, filtered, and trypsin-digested. Peptides were extracted using 2.5% formic acid in 50% acetonitrile, supplemented with 0.001% polyethylene glycol [24], and concentrated via SpeedVac.

Samples were analyzed using an UltiMate 3000 RSLCnano system coupled to a timsTOF Pro HT mass spectrometer. Peptides were online-desalted on a trapping column (Cat. No. 174500, Thermo Fisher Scientific) using 0.1% trifluoroacetic acid. Separation was performed on an Aurora analytical column (Cat. No. AUR3-25075C18-CSI; Ion Opticks) using a 60 min linear gradient (3–42% mobile phase B; flow 150 nL/min). Ionization was achieved via a CaptiveSpray source at 50°C.

Data were collected in data-independent acquisition (DIA) mode (m/z 100–1700; 1/k0 0.6–1.4 V s cm^−^²). dia-PASEF used a 21-Th window scheme (precursor range m/z 400–1000), with two steps per PASEF scan and a 100-ms cycle time (100% duty cycle).

DIA data were processed in DIA-NN (v2.2.0; [25]) in library-free mode against the cRAP, *Oryctolagus cuniculus*, and *Treponema pallidum* databases. Search settings and MBR were configured according to our previously published protocol ([22]).

Protein MaxLFQ intensities from DIA-NN were processed via a containerized KNIME workflow (v4.7.7a; [github.com/OmicsWorkflows/KNIME_docker_vnc]) involving (a) filtering of contaminants and low-quality precursors; (b) loessF-based intensity normalization; (c) protein group quantification via the iq R package; and (d) sample ratio calculation*.*

### Analysis of proteomes

Despite the descriptive nature of the proteomic data, which were obtained from a single biological replicate per strain, this dataset enables an initial comparative analysis of proteomes across the studied strains. MS/MS spectra were searched against strain-specific protein databases derived from the following NCBI assemblies: DAL-1 (GCA_000246815.1), SS14 (GCA_000410555.1), CDC-2 (GCA_000246775.1), Fribourg-Blanc (GCA_000387485.1), and Bosnia A (GCA_000813285.1). To facilitate cross-strain comparison, proteins were mapped using locus tags from genome annotations, ensuring that orthologs could be identified independently of strain-specific nomenclature. For each individual strain, the total number of proteotypic peptides (i.e. peptides mapping uniquely to a single protein) was determined, followed by the identification of corresponding proteins. Venn diagrams were subsequently used for an comparison of shared and specific protein profiles among treponemal strains or subspecies.

Due to the single-replicate design, the analysis of relative protein abundances was also kept exploratory. Normalized MaxLFQ intensities were log₂-transformed, and protein intensity distributions were compared across all analyzed strains. After excluding proteins with missing values, the 50 proteins exhibiting the highest variance between the TPE/TEN and TPA groups were selected. Principal component analysis (PCA) was utilized for evaluation of strain clustering and visualization in R (R Core Team, Vienna, Austria) using packages readxl [26], ggplot2 [27], FactoMineR [28], and factoextra [29].

### Statistical analysis

An unpaired two-tailed Student’s t-test was used to compare the multiplication rates of TPE strains during long-term cultivation. *p*-values lower than 0.05 were considered statistically significant and are denoted with asterisks according to statistical significance (∗*p* < 0.05, ∗∗*p* < 0.01, and ∗∗∗*p* < 0.001). GraphPad Prism 10 software was used for calculations.

## Results

### TPE strains can be continuously cultivated *in vitro*

While various TPA strains are now routinely cultivated *in vitro* by multiple research groups [8,11,30–34], successful *in vitro* growth of the TEN strain Bosnia A has been reported by only a single study to date [9]. Notably, the same conditions did not support the growth of the TPE strains Gauthier and Samoa F [9]. In the present study, we utilized the established *in vitro* cultivation system to verify the growth of the Bosnia A strain and, for the first time, to evaluate this system for the cultivation of three TPE strains (Gauthier, CDC-2, and Fribourg-Blanc) derived from frozen rabbit testicular stocks (Supplementary Table S1).

Dark-field microscopy of the cryostocks revealed varying treponemal densities, specifically 51.0 × 10^6^ cells/mL for Gauthier, 6.8 × 10^6^ cells/mL for CDC-2, and 0.68 × 10^6^ cells/mL for Fribourg-Blanc. Notably, all strains exhibited markedly reduced viability (approximately 10% of motile cells) immediately after thawing. The initial inoculation of motile treponemes for the *in vitro* cultures were estimated at 255,000 cells for Gauthier, 34,000 cells for CDC-2, and 3,400 cells for Fribourg-Blanc. In contrast, the inoculation dose for the TEN strain Bosnia A was significantly lower; since only one treponeme was found per 120 fields by DFM, the initial inoculation dose was estimated at approximately 15 cells.

At the initiation of cultivation, an enrichment protocol previously optimized for the isolation of treponemes from clinical samples was employed [12], as a limited number of viable treponemes was anticipated in the initial stocks. The TEN strain Bosnia A and the TPE strains CDC-2 and Fribourg-Blanc were successfully enriched *in vitro*, and following several subcultures, all three strains reached a phase of stable, long-term growth (Figure 1). During the stable cultivation phase, inoculum volumes were adjusted to 1–2 mL, representing dilution ratios of 1:3–1:6. Under this setup, all three strains were successfully maintained for over one year, reaching cumulative generations of 147, 149, and 112 for Bosnia A, CDC-2, and Fribourg-Blanc, respectively.

**Figure 1.**
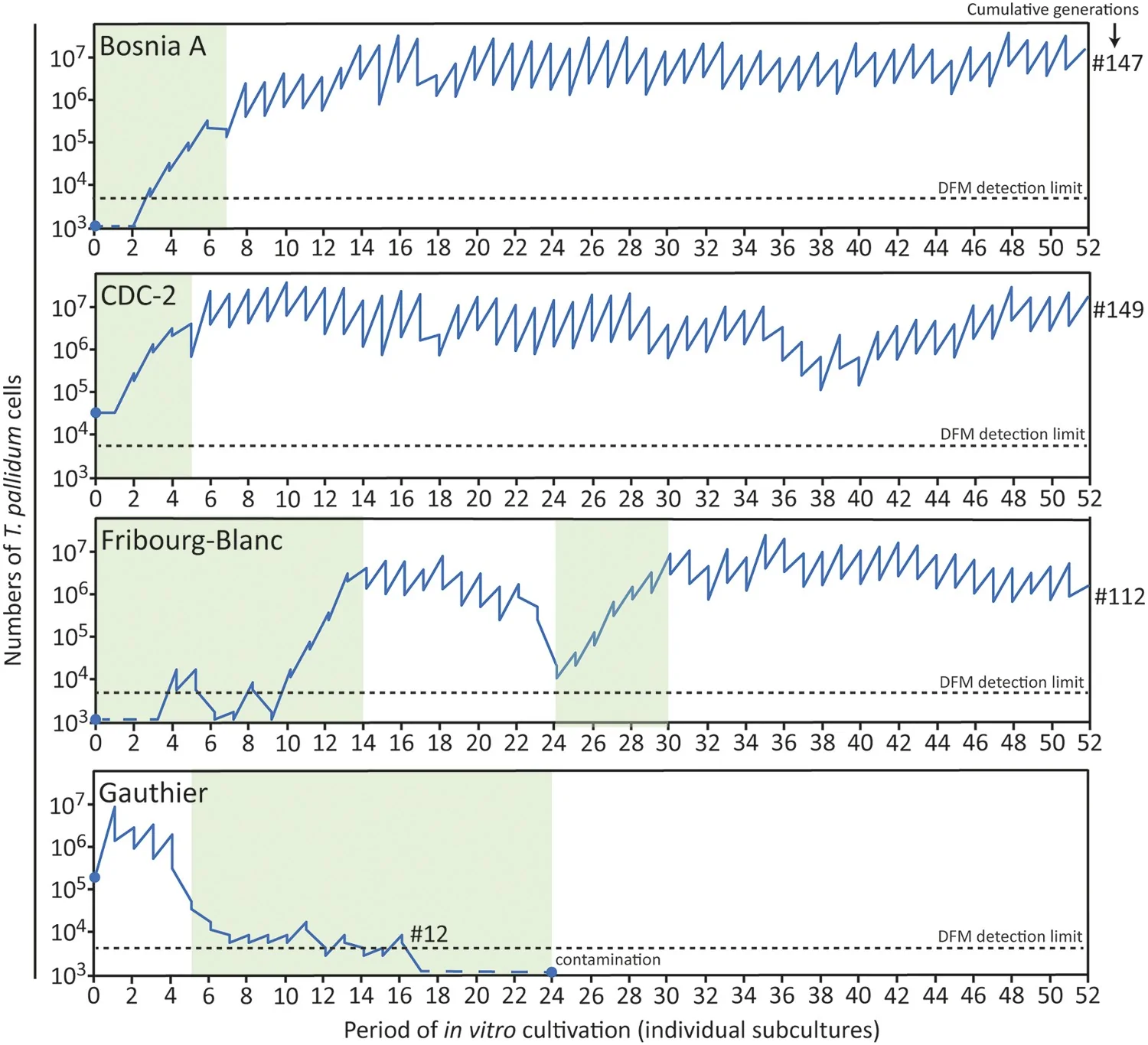
**Long-term *in vitro* cultivation dynamics of TEN and TPE strains.** The sawtooth plots represent 52 consecutive subcultures for the Bosnia A strain (TEN) and three TPE strains (CDC-2, Fribourg-Blanc, and Gauthier). Each trough and peak represents the mean treponemal counts at inoculation and harvest per passage (determined from two independent wells). While Bosnia A, CDC-2, and Fribourg-Blanc were successfully established in long-term culture, the Gauthier strain exhibited a progressive decline. The enrichment phase is highlighted in green, while the stable cultivation phase is shown in white. Cumulative generations achieved for each strain are indicated on the right.

In contrast, the TPE strain Gauthier exhibited considerably worse growth *in vitro*. Despite the highest initial inoculation dose (255,000 cells), the number of treponemes decreased progressively over the 16 weeks (Figure 1), corresponding to an estimated number of 12 cumulative generations. Although no treponemes were detectable by microscopy after this period, nested PCR confirmed the presence of Gauthier for additional eight passages, at which point cultivation was discontinued after a total of 24 weeks due to mould contamination. These results suggest that while the Gauthier strain is capable of *in vitro* multiplication, its growth rate under the tested conditions was insufficient to achieve long-term stable cultivation.

### TPE strains exhibit distinct *in vitro* growth rates

Growth rates were quantified and compared using stable subcultures from continuous cultivations of the TPE strains CDC-2 and Fribourg-Blanc, and the TEN strain Bosnia A (Figure 2A). While the CDC-2 strain showed a multiplication rate comparable to Bosnia A, with a mean weekly increase of 8.15-fold, the Fribourg-Blanc strain grew significantly slower, reaching only 5.92-fold (*p* < 0.05). Median generation times were 57.5 h for Bosnia A, 60.7 h for CDC-2, and 73.7 h for Fribourg-Blanc. Following the method of Edmondson et al. [8], minimal generation times were calculated based on the five subcultures with the highest fold increase. These values reached 40.3 h for CDC-2 and 51.6 h for Fribourg-Blanc, representing a substantial reduction compared to the overall median values.

**Figure 2.**
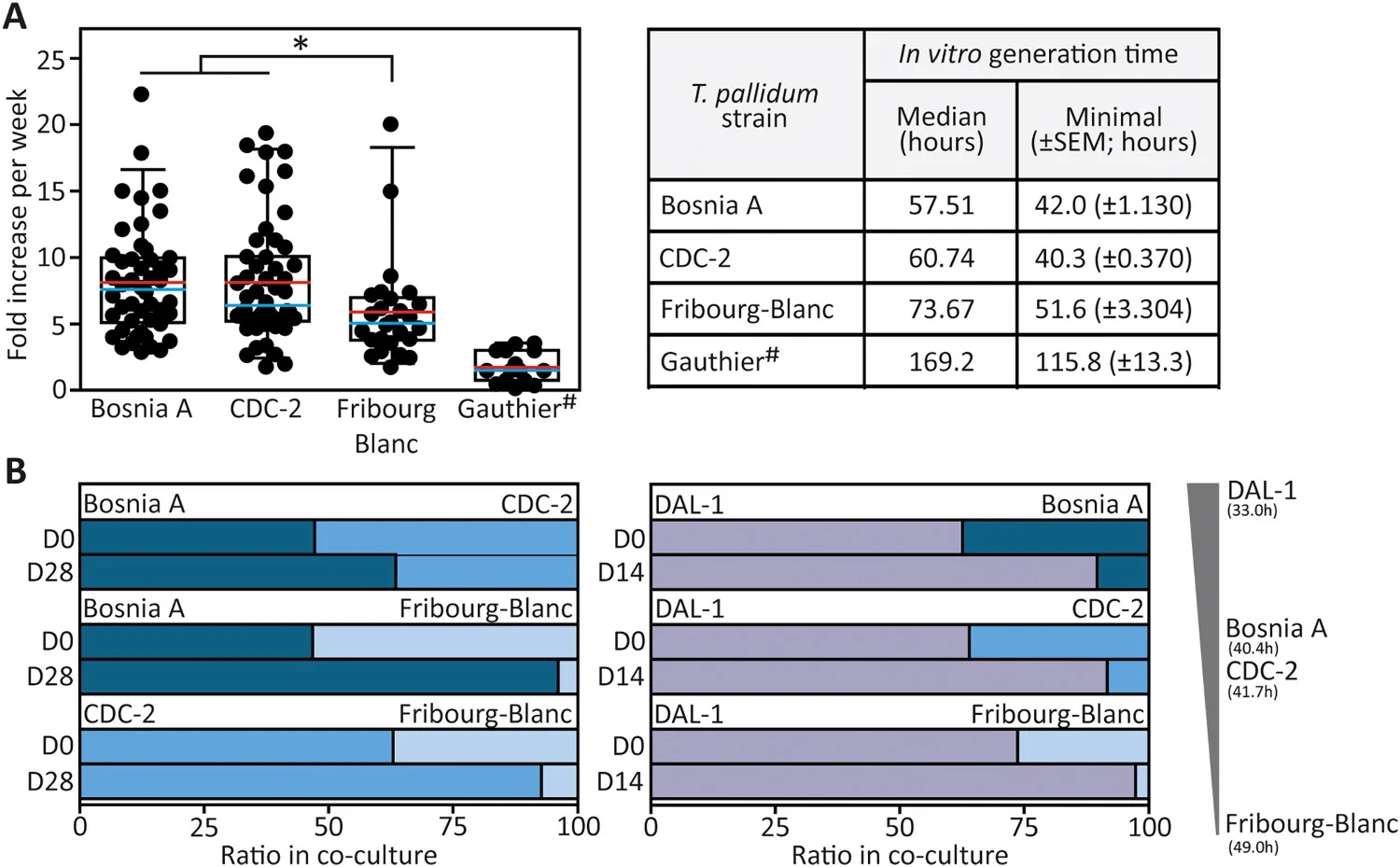
**Quantification of *in vitro* growth of TPE and TEN strains. (A)** Fold increase from standardized subcultures during long-term cultivation revealed growth differences (black circles, mean of two independent wells; red bar, mean; blue bar, median; Box and Whisker with 90% confidence interval). The simian isolate Fribourg-Blanc grew significantly slower than both CDC-2 and Bosnia A (unpaired Student t-test, *p < 0.05). #Data for the Gauthier strain were obtained under “enrichment” conditions; therefore, these results are not directly comparable with the standardized cultivation of other strains. Moreover, the estimated generation time for Gauthier is likely affected by these suboptimal growth conditions. **(B)** Multiplication of TPE and TEN strains in binary co-cultivations. Slower *in vitro* growth of Fribourg-Blanc was confirmed (left). Generation times for TPE and TEN strains were derived using the TPA strain DAL-1 as a reference (33.0 h; [11]) (right). The obtained values correspond closely to the minimal generation times observed during long-term cultivation. Co-cultivation with DAL-1 was evaluated after two weekly subcultures due to fast overgrowth by DAL-1, precluding reliable analysis at later time points. The experiment was performed in one biological replicate. Detailed quantification data are provided in Supplementary Table S4.

To verify the observed growth differences and minimize the impact of potential variability between individual subcultures, the TPE strains (CDC-2 and Fribourg-Blanc) were independently co-cultivated with the TEN strain Bosnia A (see Methods for details). Overall, the growth results in these co-cultivation experiments were in accordance with the results from the continuous cultivation (Figure 2B, left). The CDC-2 strain exhibited growth comparable to Bosnia A, whereas Fribourg-Blanc showed a consistently lower multiplication rate. To calculate generation times, CDC-2, Fribourg-Blanc, and Bosnia A were individually co-cultivated with the TPA strain DAL-1, which served as a reference strain with a defined *in vitro* generation time of 33.0 h [11]. All three strains grew considerably slower than DAL-1, and their generation times derived from co-cultivation with DAL-1 (Figure 2B, right) corresponded closely with the minimal generation times observed in long-term cultivation. The internal consistency across co-cultivations, combined with the lack of detectable growth antagonism, indicates that the identified differences are inherent to the strains themselves.

### Proteomic profiles of CDC-2, Fribourg-Blanc, and Bosnia A

Successful *in vitro* cultivation of CDC-2, Fribourg-Blanc, and Bosnia A allowed the first evaluation of their proteomes. Proteomic profiling identified a substantial portion of the predicted treponemal proteins across analyzed strains (Figure 3A). For the TEN strain Bosnia A, we detected 889 proteins (representing 83.6% of its annotated proteins), with 813 of these identified by two or more unique peptides. The TPE strains yielded slightly lower coverage, with 776 proteins detected for CDC-2 (72.7% of annotated proteins) and 694 for Fribourg-Blanc (65.2% of annotated proteins), including 670 and 596 proteins identified by at least two peptides, respectively. These variations in coverage directly correspond to the differences in initial cell counts (Bosnia A: 1.7 × 10^6^, CDC-2: 0.4 × 10^6^, and Fribourg-Blanc: 0.13 × 10^6^). Detailed correlation between input material and peptide recovery is provided in Supplementary Figure S1.

**Figure 3.**
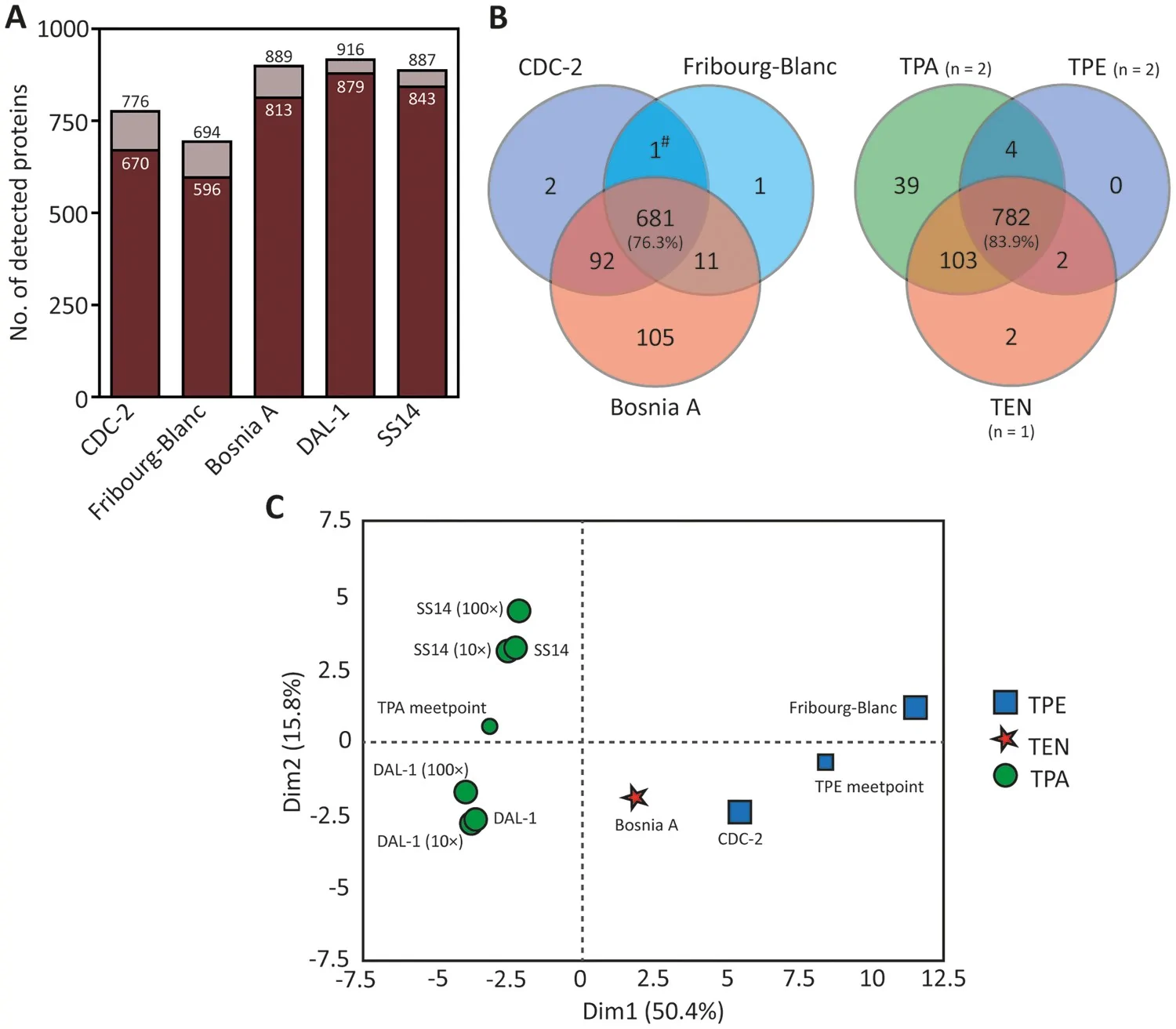
**Analysis of T. pallidum proteomes. (A)** High-quality proteomes were recovered for TPE, TEN as well as for TPA subspecies. Columns represent the total number of detected proteins for individual strains (CDC-2, Fribourg-Blanc, Bosnia A, DAL-1, and SS14). The darker segments indicate the portion of proteins identified by two or more unique peptides. Detailed peptide counts are provided in Supplementary Figure S1A. **(B)** Comparison of proteomes across treponemal strains and subspecies. Venn diagrams illustrate the overlaps among CDC-2, Fribourg-Blanc, and Bosnia A (left), and among the three *T. pallidum* subspecies (right). While the majority of detected proteins form a core proteome, several proteins were observed as specific to individual strains or subspecies, or shared by only a subset of groups, however, these findings remain exploratory (1 biological replicate with lower coverage in TPE proteomes). #TP0129 was not annotated in the Bosnia A genome. **(C)** Exploratory analysis of quantitative differences. The PCA indicates a separation among the TPA, TPE, and TEN subspecies. Diluted TPA samples cluster with their undiluted counterparts, which supports the observed separation. PCA analysis was performed based on the 50 most contributing proteins; see Supplementary Figure S1B for the PCA based on all detected proteins.

A comparison of the proteomes revealed that CDC-2, Fribourg-Blanc, and Bosnia A share 681 detected proteins (76.3% of the identified proteome). While Bosnia A contained 105 strain-specific proteins, only two were unique to CDC-2 (TP0716, *fli*R; TP0843, hypothetical protein) and one to Fribourg-Blanc (TP0603, *upp*S) (Figure 3B, left). When proteomes of TPA strains DAL-1 and SS14 (analyzed in parallel with the TPE and TEN proteomes to minimize technical variability, Figure 3A) were included in the analysis, a total of 932 treponemal proteins were detected, revealing a core proteome of 782 proteins shared across *T. pallidum* subspecies (83.9%; Figure 3B, right). Comparative analysis identified 103 proteins shared exclusively between TPA and TEN, whereas only 4 were shared between TPA and TPE, and 2 were shared by TPE and TEN. However, due to the single biological replicate and lower coverage in TPE proteomes, this analysis should be regarded as exploratory rather than reflecting true biological differences.

Furthermore, an exploratory analysis of quantitative differences among the obtained proteomes of various *T. pallidum* subspecies was performed using Principal Component Analysis (PCA). For a comparison independent of proteome depth, the analysis was restricted to the 50 most contributing proteins, defined as those showing maximum quantitative differences between TPE/TEN and TPA proteomes. The PCA indicated a separation of TPA, TPE, and TEN subspecies (Figure 3C). Additionally, 10- and 100-times diluted samples of the high-input TPA strains (see Methods) were included to test the robustness of the clustering. Diluted TPA samples clustered closely with their undiluted counterparts, indicating that the separation does not reflect variability in sample concentration, but rather subspecies/strain-specific differences.

The full list of detected proteins and their parameters of identification is provided in Supplementary Table S5.

## Discussion

While *in vitro* cultivation of TPA strains is successfully maintained by various research groups [8,11,30–34], *in vitro* data for non-TPA subspecies remain extremely limited. To date, the only other reported successful *in vitro* cultivation involved the TEN Bosnia A strain [9]. Our results verified the *in vitro* growth of Bosnia A with parameters highly consistent with the previous report, as both studies estimated a generation time of approximately 42 h.

This study provides the first successful long-term *in vitro* cultivation of two TPE strains. This included the CDC-2 strain, originally isolated from a human yaws patient in Africa [Akorabo, Ghana, 1980], and the Fribourg-Blanc strain, derived from an infected African baboon (*Papio papio*) [Guinea, 1966]. The identity of successfully maintained *in vitro* cultures was confirmed by sequencing of specific DNA loci (TP0136, TP0548). Moreover, proteomic analyses identified sequence-unique peptides specific to CDC-2 (n = 8) and Fribourg-Blanc (n = 6), which further confirmed the respective strain identities (data not shown).

The successful long-term *in vitro* cultivation of TPE strains was achieved by implementing two strategies from our previous work. First, we utilized an enrichment step, originally designed for isolation of TPA from clinical samples [12], to accumulate sufficient treponemal numbers from a limited inoculum. This step can also be applied in cases where cultures face suboptimal conditions. Second, rather than the high dilution ratio of 1:20 originally described for the TPA Nichols strain [8], we used a significantly larger inoculum (i.e. ratios of up to 1:3) during subculturing, a modification previously validated for slow-growing TPA strains [11]. The combination of an effective enrichment approach and optimized, high-density passaging enabled the long-term maintenance of slow-growing TPE strains.

In our previous study, generation time of TPA strains ranged from 33.0 h (DAL-1) to 43.5 h (Philadelphia 1) [11]. Here, the CDC-2 strain showed a generation time of 41.7 h, which is comparable to Bosnia A (40.4 h) and the slowest-growing TPA strains. Notably, the Fribourg-Blanc strain reached 49.0 h, representing the longest *in vitro* generation time recorded to date. Furthermore, the generation time of the Gauthier strain is likely even longer.

While both CDC-2 and Fribourg-Blanc were successfully maintained for over a year, the Gauthier strain exhibited restricted *in vitro* growth. Although it remained viable and capable of multiplication for at least 16 weeks, its limited growth dynamics precluded long-term cultivation under the tested conditions. This growth limitation was unlikely caused by a suboptimal inoculum size or cryopreservation-associated damage. In fact, Gauthier had the highest initial inoculum and successfully maintained high counts during the initial four weeks (see Figure 1). Furthermore, since the cultivation system utilizes rabbit-derived cells, attenuation through prolonged rabbit passage does not explain the observed growth limitation. Instead, we hypothesize that the limited growth of the Gauthier strain reflects inherent genetic characteristics affecting its *in vitro* fitness.

Similar results were reported by Edmondson et al. [9], who maintained Gauthier (and Samoa F) *in vitro* for only two weeks, suggesting an inherent fitness limitation that prevents long-term maintenance under current *in vitro* conditions. Such growth differences among TPE strains are plausible, given the fact that *in vitro* growth variations were already documented within TPA strains [11], and the relatively high genomic diversity of TPE [14,35] could predispose these strains to broader fitness variations. This is further supported by recent evidence showing that single nucleotide polymorphisms can directly alter treponemal growth rates [36]. However, further studies will be needed to elucidate the genetic basis of *in vitro* growth differences.

Our study demonstrates that TPE strains are cultivable *in vitro*, but cultivation efficiency varies among strains, which indicates that current protocols need to be optimized for Gauthier and other strains with specific requirements. To achieve stable long-term growth, we hypothesize that outcomes could be improved through the reduction of treponemal loss during subcultivation and the extension of passage intervals for slow-growing strains. Although several human cell-based cultivation systems have been recently established for TPA strains, none have achieved significantly better support for treponemal growth [37–39]. Since rabbit systems remain the standard for both *in vivo* and *in vitro* cultivation, the specific origin of feeder cells does not appear to be critical. Instead, research should focus on identifying or developing cell lines, regardless of origin, with intrinsically slow growth rate. This feature appears crucial for successful *in vitro* culture [37,38], likely by minimizing nutrient competition within the cultivation well.

Successful *in vitro* cultivation allowed for the first-time proteome profiling of TPE (CDC-2, Fribourg-Blanc) and TEN (Bosnia A) strains, which significantly expands the limited number of existing proteomic studies that have so far targeted only TPA strains [22,30,40–43]. Although this initial characterization is based on a single replicate with differences in proteome coverage and remains qualitative, it serves as a valuable baseline dataset. Further independent measurements and the analysis of more treponemal strains, together with detailed quantitative profiling will be required in future studies to identify strain- and subspecies-specific markers associated with the pathogenesis of syphilis, yaws, and bejel.

Besides the abovementioned descriptive character of the proteomes, there are some other limitations that need to be discussed. First, the observed slower growth of TPE and distinct growth rates among individual TPE strains may partly reflect their long-term laboratory history. These strains were isolated decades ago with unknown propagation records in rabbits and hamsters, raising the possibility of adaptation to animal models. To verify the true growth parameters of the yaws and bejel strains, future studies must evaluate contemporary TPE and TEN isolates with minimal animal passages. To our knowledge, no contemporary TPE isolates are currently available. Nevertheless, an animal-free *in vitro* protocol for the isolation of TPA strains was recently introduced [12], and its extension to TPE and TEN is expected. Another important limitation is that this study did not verify the pathogenicity of TPE strains after long-term *in vitro* cultivation. However, a loss of virulence is unlikely, as previous studies have demonstrated that TPA strains remain fully virulent after several years of continuous *in vitro* propagation [8,32].

TPE strains share over 99.8% genomic identity with TPA [1,2,44] and are morphologically and serologically indistinguishable [45], leaving molecular diagnostics as the only discriminative method [46]. Nevertheless, biological differences exist in invasiveness, pathogenicity, and transmission routes [3]. Our study fills the gap by the introduction of successful *in vitro* cultivation of TPE strains, which for a long time remained uncultivable despite the established cultivation of TPA. We achieved this for both human and non-human primate strains, which reflects the fact that human and animal TPE strains are not genomically distinct [14,35]. Furthermore, the observed slower *in vitro* growth may represent another biological difference between TPA and TPE subspecies.

Taken together, this study demonstrates successful long-term *in vitro* cultivation of TPE strains, which provides an important tool to investigate yaws pathogenesis and non-human primate infections.

## Supplementary Material

Supplementary Table S5.xlsx

Supplementary Table S2.xlsx

Supplementary Table S3.xlsx

Figure_S1.docx

Supplementary Table S1.xlsx

Supplementary Table S4.xlsx

## Data Availability

The mass spectrometry proteomic data have been deposited to the ProteomeXchange Consortium via the PRIDE partner repository with the dataset identifier PXD078905. The sequencing data have been submitted to the NCBI BioProject database under the accession number PRJNA1470989. All other data generated or analyzed during this study are included in this published article and its supplementary files, or are available from the corresponding author upon reasonable request.
