# Supplementary material for: Successful long-term in vitro cultivation of Treponema pallidum subsp. pertenue strains of human and non-human primate origin
Source: Emerg Microbes Infect. 2026 Oct 17;15(1):2751730. doi: 10.1080/22221751.2026.2751730 (PMC13757798; doi:10.1080/22221751.2026.2751730)
Supplement: Supplementary file 1 [file TEMI_A_2751730_SM5067.docx]

**Figure S1.** **Additional data for the proteomic analysis. (A)** Detailed peptide and protein counts, and their correlation with the input material across the studied strains. **(B)** Principal component analysis (PCA) of *T. pallidum* strains using intensities from the complete set of detected proteins. Although the clusters are less compact than those in Fig. 3C, a distinct separation of the groups remains clearly visible even when using the full proteome dataset.
